# Developing the public health workforce: training and recognizing specialists in public health from backgrounds other than medicine: experience in the UK

**DOI:** 10.1186/s40985-018-0091-x

**Published:** 2018-06-15

**Authors:** Selena F. Gray, David Evans

**Affiliations:** Department of Health and Social Sciences, University of the West of England, Bristol, Room 3L15C, Bristol, BS16 1QY UK

**Keywords:** Public health workforce, Capacity development, Professional regulation

## Abstract

**Background:**

There is increasing recognition that improving health and tackling inequalities requires a strong public health workforce capable of delivering key public health functions across systems. The World Health Organization in Europe has identified securing the delivery of the Essential Public Health Operations and strengthening public health capacities within this as a priority.

It is acknowledged that current public health capacities and arrangements of public health services vary considerably across the World Health Organization in European Region, and investment in multidisciplinary workforce with new skills is essential if public health services are to be delivered.

**Case presentation:**

This paper describes the current situation in the UK where there are nationally funded multidisciplinary programmes for training senior public health specialists. Uniquely, the UK provides public health registration for multidisciplinary as well as medical public health specialists.

**Conclusion:**

The transition from a predominantly medical to a multidisciplinary public health specialist workforce over a relatively short timescale is unprecedented globally and was the product of a sustained period of grass roots activism aligned with national policy innovation. the UK experience might provide a model for other countries seeking to develop public health specialist workforce capacity in line with the Essential Public Health Operations.

## Background

There is increasing recognition that improving health and tackling inequalities requires a strong public health workforce capable of delivering key public health functions across systems. WHO Europe have identified securing the delivery of the Essential Public Health Operations (EPHOs) (Table 1) and strengthening public health capacities within this as a priority [1].

**Table 1:** Ten essential public health operations (EPHOs)

1. Surveillance of population health and well-being	
2. Monitoring and response to health hazards and emergencies	
3. Health protection including environmental, occupational, food safety and others	
4. Health promotion including action to address social determinants and health inequity	
5. Disease prevention, including early detection of illness	
6. Assuring governance for health and well-being	
7. Assuring a sufficient and competent public health workforce	
8. Assuring sustainable organizational structures and financing	
9. Advocacy, communication, and social mobilization for health	
10. Advancing public health research to inform policy and practice	

Source: WHO Regional Office for Europe. European Action Plan for Strengthening Public Health Capacities and Services. Copenhagen: WHO; 2012

It is acknowledged that current public health capacities and arrangements of public health services vary considerably across the WHO European Region and investment in a multidisciplinary workforce with new skills is essential if public health services are to be delivered [1]. This report notes that “significant efforts are required to scale up not only the number of public health professionals, but also their quality and relevance to public health” (p. 17, para 21) and recommends that regulation and accreditation mechanisms should be supported (para 73). Foldspang has argued that we should move (in Europe) towards shaping an authorized profession based on public health competencies and the EPHOs that should encompass agreed standards and ethics [2]. In a review of four countries in Europe (France, Portugal, UK, and Poland), Foldspang and Otok concluded that “The United Kingdom has got the most comprehensive and coherent public health system and a well-suited framework for the development and maintenance of a competent public health workforce, with participation from public health professionals with other than medical training, and well organised in comprehensive professional associations” [3].

Whilst WHO, as described above, has identified key public health operations, others have attempted to define a system of core competences which could be applicable to what is deemed to be higher level practice in public health in the key areas of methods in public health; population health and its determinants; health policy; economics; organizational theory and management; health promotion; health education, health protection and disease prevention, and ethics [4].

This paper will highlight the current situation in the UK with respect to training of public health specialists—i.e. those who are expected to take up senior leadership roles in public health in a variety of settings, the history of this development, and key factors in its implementation.

## Case presentation

### Where we are now?

The current position in the UK is that there are nationally funded programmes for training senior public health specialists who will be expected on completion of training to take up senior roles in different parts of the public health system in the UK. These are normally of 5 years duration, with the first year undertaking a Master’s level qualification in Public Health. Subsequently, individuals undertake a competency-based training working in a variety of different settings, completing professional exams and workplace-based assessments. Entry is highly competitive and is open to individuals from any background who can demonstrate a relevant degree and experience. Doctors are able to enter following the first 2 years of practice after registration, whilst others are required to have some experience in a public health setting. The UK Faculty of Public Health, a professional body, has responsibility for developing the curriculum and setting the exams; the final curriculum is approved by the regulator who holds the register of individuals who have completed the training and are therefore able to demonstrate professional capabilities in public health practice. For doctors, the General Medical Council (GMC) is the regulator and holds this register; applicants are on a specialist register in public health medicine. For those with a background other than medicine, the UK Public Health Register (UKPHR) holds that list. The UKPHR is a charity and company limited by guarantee. (This is the same process for all recognized medical specialties, with professional bodies of colleges and faculties working closely with the regulator, the GMC).

There are also processes for individuals who have not gone through a formal training programme but can demonstrate that they have acquired the relevant substantial experience to gain entry to both the UKPHR and GMC registers through a portfolio type route. (These processes are currently being harmonized between the two regulators). Individuals taking up key public health leadership roles within the system are expected to be on a register to be eligible for appointment.

In 2012, there were an estimated 1100 public health specialists working in England [5]. Since 2013, the public health function in England is predominantly located within local authorities and a national organization, Public Health England. Of those, 574 individuals responded to a survey that found that 53% worked for local authorities, while 30% worked for Public Health England, and 13% within universities. Approximately half were registered with the GMC and half with UKPHR. Two thirds had completed specialty training and 29% had qualified via the portfolio route. However, the proportion coming onto the register from training programmes will increase with time—the majority of individuals gaining access to registration in 2016 coming from training programmes [5]. The UK Faculty of Public Health reported that in 2016/2017, there were six candidates admitted to the UK Public Health Register by the training and examination route, four by the portfolio route, and nine onto the GMC register [6]. Over the last few years, the proportion of those entering public health training has remained stable with approximately 37% from medicine and 63% from other backgrounds [7].

### How did we get here?

The transition from a predominantly medical to a multidisciplinary public health specialist workforce over a relatively short timescale is unique globally and was the product of a sustained period of grass roots activism aligned with national policy innovation. The story of its origins has been told [8–11] but is worth summarizing those accounts and bringing them up to date here. In the 1990s, a number of public health practitioners were increasingly frustrated by the “glass ceiling” on their public health careers, whilst far-sighted public health professional leaders realized that public health was intrinsically a multidisciplinary endeavor and that developing public health specialist capacity required drawing on a range of professional backgrounds. This movement found a receptive response in politicians and policy makers who were ideologically inclined to support the breaking down of professional barriers.

From 1997, a “Tripartite Group” made up of the activist Multidisciplinary Public Health Forum, the Royal Institute of Public Health, and the Faculty of Public Health Medicine worked with the English Department of Health to develop new professional structures, in particular for training and regulation, to enable the recognition of non-medical public health specialists. Despite some opposition from elements of the public health medicine specialty, a number of parallel and complementary policy changes were enacted. In 1999, a government white paper committed to develop a new non-medical role of specialist in public health [12]. From 2000, the Faculty of Public Health Medicine took the critical steps of opening up its examinations, membership, and fellowships to qualified candidates from any professional background. At around the same time, the English regional training programmes for public health specialists were opened to applicants from different backgrounds, initially on a more limited basis but over time increasingly with a common application and entry processes for medical and non-medical candidates.

The need to ensure the standards for multidisciplinary specialist public health was a key concern of the Tripartite Group from the beginning; following an encouraging statement from a government minister in 2001 indicating support for a “voluntary register”, the Tripartite Group began developing plans to make such a register a reality. There were many discussions and debates along the way, particularly over assessment criteria and on whether to “grand-parent” in existing senior non-medical public health professionals. With the support of the Faculty of Public Health (medicine having been dropped from the name to reflect the new multidisciplinary nature of the organization) and funding from the Department of Health, the new UK Voluntary Register for Public Health Specialists (later UKPHR) was launched in March 2003.

Initially there were two routes for specialist registration with UKPHR. For those existing public health professionals with substantive experience, there was the opportunity to submit for portfolio assessment; this included the first cohort of non-medical directors of public health from 2002 who were offered provisional registration even before submitting their portfolios on the understanding that they would submit within 2 years. The other “standard” training route was open to those who completed the recognized specialist training programme. Initially, the scope and length of this training for non-medical trainees varied between 3 and 5 years, but increasingly it was regularized to a common 5-year public health registrar programme alongside medical trainees. The first non-medical trainees began their programmes around 2001, so it was some years before significant numbers had completed their training and were able to register with UKPHR.

In the early days of the UKPHR, huge efforts were made to ensure the credibility of the register and its processes with key stakeholders, in particular the health departments of the devolved administrations in the four UK countries, FPH, GMC, employers, and of course potential registrants. Close links were maintained for example with the FPH to ensure that those registered on the UKPHR worked to similar standards and CPD requirements to those for specialists registered with the GMC. The willingness of employers to specify UKPHR registration as a criterion for specialist employment was crucial. The success of the initiative was determined by the increasing number of non-medical public health professionals applying for registration.

One continuing debate focused on whether registration should move from a voluntary to a statutory basis and if so, what the registration body should be. A report commissioned and published by the Department of Health in 2010 recommended that there should be statutory registration through the Health Professions Council rather than the UKPHR [13]. This proposal was included in the Public Health Workforce Strategy in 2013 [14] and apparently confirmed following a period of consultation in 2014–2015, before being unexpectedly postponed indefinitely by the Department of Health in 2016 [15]. This unresolved debate has not, however, appeared to damage the continuing appeal of UKPHR registration to public health specialists from backgrounds other than medicine.

### What helps make it a success?

Some of the features that have been critical to the success of the integrated training programmes have been common entry standards, a common curriculum and assessment standards for training programmes, and the almost universal adoption by employers of a requirement for registration as a public health specialist with the GMC or UKPHR within job descriptions and appointment processes.

Historically, recruitment to speciality training programmes was undertaken at local level, but in 2009, a national recruitment and selection process was introduced [7]. This consisted of a two-stage competency-based process, explicitly linked to a detailed person specification. The first stage, assessment centre (AC), is comprised of two cognitive ability tests, which measure numerical (Rust Advanced Numerical Reasoning Appraisal test) and verbal reasoning (Watson Glaser Critical Thinking test). A situational judgment test developed specifically for use in the public health context was added in 2011. Progression to the second stage, selection centre (SC), requires applicants to pass the threshold score for each of the three tests and those with the highest combined scores are invited to the selection centre. The SC has three components: a group exercise, a written test, and a series of short interview panels. This rigorous and competitive entry process has ensured consistently high standards across all those entering training programmes irrespective of their professional background.

From the beginning, there were single integrated training programmes for those from all backgrounds, with common exams, curricula, and assessment frameworks, agreed and supported by both regulators and leading to registration with the GMC for doctors, and UKPHR for those with a background other than medicine. Although the training routes have been identical, the portfolio assessment route for the two regulators has differed, leading to some concerns about consistency of standards; however, this is about to change with UKPHR issuing proposals to align the portfolio assessment route to the single curriculum [16].

There was undoubtedly been a strong political will and commitment to the development of a multidisciplinary workforce, as outlined in key policy documents at the time [12]. Without this support to expand the opportunities for public health leadership roles to be open to those from all backgrounds, it would have been difficult to have made progress. Another key factor was ensuring that the requirement for all public health specialists to be on the UKPHR or GMC register was embedded as a requirement within all job descriptions for senior public health posts; whilst this was relatively straightforward for national organisations such as Public Health England, it has required more vigilance to embed this as a requirement within local authorities and has required an active partnership between policy makers and professional bodies to maintain standards in the field.

### What are the barriers and risks?

It is tempting to suggest that there are no significant barriers to developing multidisciplinary public health specialist capacity, given the undoubted success that has been achieved in the UK over the last 20 years. But that would be to diminish and fail to acknowledge the activism, innovation, and hard work that has led to so many barriers being overcome. Chief among the early barriers which largely have been surmounted was the understandable concern by some public health doctors that opening public health specialist status to non-doctors would diminish the status of the specialty and/or lead to their gradual replacement by cheaper non-medical alternatives. Although these concerns may have diminished over time, they have not entirely disappeared.

The contemporaneous transfer of the public health function from the English NHS to local government [1] in 2013 has potentially exacerbated such concerns, with public health doctors tending to migrate towards roles in the civil service within Public Health England whilst local government roles have more commonly been taken by specialists from backgrounds other than medicine. There is some evidence that this is happening as the most recent data on the specialist public health workforce in England [17] as of March 2017 which shows that whilst the overall numbers of specialist have risen by 3% since 2015 to a total of 1170 (965) FTE specialists and Directors of Public Health employed in local authorities, PHE, the NHS, and universities, numbers have increased by 11% in PHE, the NHS and universities, but have fallen by 5% in local authorities since 2015; 55% are registered with the General Medical Council. There is some variation between sectors, with local authority staff more likely to be female, aged under 50, and from backgrounds other than medicine and PHE/NHS/university staff more likely to be male, over 50 and from a medical background.

These tendencies risk reestablishing and deepening a division between medical and social models of public health each in their own professional “silo”. Alongside this risk is a concern that financially pressed local authorities may seek to employ non-medical public health specialists on lower salaries and poorer terms and conditions than medical specialists would receive. With the autonomy that local authorities enjoy, there is a further risk that they may seek to employ fewer specialists or cease requiring those appointed to have recognized registration with the UKPHR or GMC. There are however some encouraging findings from recent graduates of the training scheme that demonstrate good progression into consultant posts with evidence that “those from backgrounds other than medicine reaching all parts of the profession” [18].

The lack of a statutory basis for the UKPHR means that it is inevitably less secure as a system than GMC registration; however, in practice the requirement for registration appears to be recognized and used by employers so this may be a theoretical rather than a substantial risk.

Finally and importantly, there is a significant difference between the portability of medical and non-medical registration. Public health doctors have a specialist status and GMC registration that enables reciprocal recognition and allows them to work globally in many countries and for international organizations; there is no similar universal recognition or status for UKPHR registration. For non-medical public health professionals in the vast majority of countries, there is no equivalent registration system to the UKPHR, and in many countries, senior posts in public health are effectively limited to medically registered public health specialists.

## Discussion

### Why is this important?

The WHO has identified that there are substantial workforce needs to deliver the Essential Public Health Functions (EPHOs) to improve population health and well-being and to reduce inequalities [1]. Given this need it is pertinent to ask how best to ensure the development of a workforce sufficiently skilled and adequately sized to deliver these functions and how appropriate or necessary it is to limit senior roles to those from a medical background. Across the European region, the Association of Schools of Public Health in Europe (ASPHER) have undertaken a substantial amount of work to develop curricula for master’s in public health courses and to map competencies to the EPHOs and to develop competencies for higher level practice in public health [4]—arguably these competencies do not require a prior background in medicine. There are also a great many undergraduate or Bachelors programmes in public health that have been developed in the European setting. What are the long-term career opportunities for these individuals? Much of the work on competencies is in parallel to, and not necessarily aligned to, the public health-orientated medical training and intern programmes in public health in different countries.

Increasingly those working in public health from backgrounds other than medicine are required to have undertaken a master’s qualification in public health; however, we would argue that a master’s level degree is an entry level qualification for public health and that additional formal training programmes are needed to support the development of future leaders in a more systematic fashion. These programmes should be integrated with the postgraduate medical/intern specialty training programmes and open to those from any backgrounds who can demonstrate the appropriate capabilities and motivation and should lead to registration as a public health specialist that would ideally be recognized by different countries and employers.

To date, it has been surprising that other national public health systems have not sought to learn from the UK experience and develop their own multidisciplinary public health specialist capacity. It is notable that there have not been any such developments even in such anglophone countries as Australia, New Zealand, and the Republic of Ireland where the public health systems have historically followed the UK model. Although anecdotally there have been some informal discussions in these countries, there have been no formal reviews or inquires nor any substantive academic or professional papers published on the potential transferability of the new multidisciplinary UK model. Indeed, there is little specific evidence on the benefits of either a medically or a multidisciplinary-led public health system as no comparative research has been done. The best evidence we currently have are the various recent national policy documents and reviews on UK public health, for example the recent House of Commons Health Select Committee inquiry into public health post 2013 which have been unanimous in their support of multidisciplinary public health specialist workforce development and leadership [19].

## Conclusion

The development of a multidisciplinary public health workforce, underpinned by common selection, curriculum, and assessment processes within an integrated training programme, which leads to registration with a statutory regulator (GMC) or a voluntary register (UKPHR) is now well established in the UK. Key issues for success have included political will, legislation, ownership by the profession, the establishment of a recognized regulator for those from a background other than medicine, common standards, and the adoption by key employers of the need for registration as a public health specialist within job specifications.

This could act as a model for the more structured and systematic development of senior leaders in public health elsewhere and provide a mechanism for career development and parity for all those working in public health irrespective of their professional background.
